# AutoPCOS: a stepwise multimodal intelligent framework for polycystic ovary syndrome risk stratification and diagnostic support

**DOI:** 10.3389/fendo.2026.1760541

**Published:** 2026-04-21

**Authors:** Jianping Hou, Yirui Duan, Wanli Zhao, Qianpeng Sun, Jiayin Wang

**Affiliations:** 1 School of Computer Science and Technology, Faculty of Electronics and Information Engineering, Xi’an Jiaotong University, Xi’an, China; 2 Autobio Labtec Instruments Co., Ltd, Zhengzhou, China

**Keywords:** clinical decision support, intelligent agent, Lingshu, machine learning, polycystic ovary syndrome (PCOS), subgroup analysis

## Abstract

**Introduction:**

Polycystic ovary syndrome (PCOS) is a prevalent endocrine disorder among women of reproductive age, typically diagnosed through a combination of clinical evaluation, laboratory testing, and ultrasonography. However, this multimodal diagnostic pathway is often time-consuming, costly, and dependent on resource availability, thereby limiting its accessibility in real-world clinical settings.

**Methods:**

In this study, we propose AutoPCOS, a stepwise multimodal intelligent framework for flexible PCOS risk stratification and diagnostic support. Using a publicly available Kaggle PCOS dataset, features were categorized into three modalities: clinical, laboratory, and ultrasound data. Based on data availability, four predictive models were constructed: (1) clinical-only, (2) clinical + laboratory, (3) clinical + ultrasound, and (4) full multimodal models. Random Forest was employed as the primary classifier, with comparisons against Logistic Regression, Support Vector Machine, Decision Tree, and Gradient Boosting. Subgroup analyses were conducted based on body mass index (BMI) and menstrual cycle patterns.

**Results:**

The proposed framework demonstrated robust predictive performance across different data availability scenarios. Notably, the models achieved strong performance in subgroups with BMI < 24 and irregular menstrual cycles, with precision values reaching ≥ 0.929. Comparative analysis confirmed the effectiveness of the Random Forest model. Furthermore, the integration of a knowledge base and the Lingshu large language model enabled interpretable risk explanations and personalized recommendations.

**Discussion:**

AutoPCOS provides a flexible and resource-aware framework for PCOS risk assessment that adapts to varying clinical conditions and data accessibility. By supporting stepwise decision-making and enhancing interpretability, the system shows potential as a practical tool for both patients and healthcare providers. Future work will focus on validation using real-world clinical datasets and improving model generalizability.

## 1 Introduction

Polycystic ovary syndrome (PCOS) is one of the most common endocrine disorders among women of reproductive age. Because no single definitive test exists, diagnosis typically follows the Rotterdam criteria (1), which require at least two of the following: ovulatory dysfunction, clinical or biochemical hyperandrogenism, and polycystic ovarian morphology (PCOM) on ultrasound. This multi-modal diagnostic pathway is time-consuming, costly, and often operator-dependent. For example, manual counting of ovarian follicles is labor-intensive and susceptible to inter-operator variability, contributing to diagnostic inconsistencies. Limited awareness and recognition of PCOS among both patients and clinicians further exacerbate the problem. Many women seek care only after experiencing infertility, by which time the syndrome may have progressed. In addition, misdiagnosis or delayed diagnosis is common when isolated symptoms are assessed without consideration of the broader syndromic pattern. Together, these challenges highlight the need for more accessible and consistent approaches to PCOS risk assessment and diagnostic support, particularly in resource-limited settings.

The rapid expansion of artificial intelligence (AI) in medicine has stimulated growing interest in its application to PCOS diagnosis (2). Within the diagnostic framework, the identification of PCOM is central (3). However, the absence of consensus regarding optimal ultrasound criteria has resulted in diagnostic subjectivity and variability (4). Consequently, numerous studies have focused on applying deep learning to improve radiologic image quality and enhance visualization of follicles, thereby increasing the accuracy and reliability of follicle counts (5, 6). Parallel work has explored machine learning and deep learning models for automated PCOM detection from ultrasound images, aiming to reduce operator dependence and provide objective, efficient diagnostic tools (7–13).

Despite the emphasis on imaging, PCOS diagnosis still relies heavily on ultrasonography and serum hormone testing—procedures that are time-sensitive and often associated with poor patient compliance. This underscores a critical need for simplified, non-invasive screening methods. Emerging evidence suggests that alterations in sex hormone metabolism and systemic circulation in PCOS may manifest through external physical signs (14–16). AI offers a powerful approach for discovering and quantifying these manifestations. For example, deep learning models, including multi-instance learning and convolutional neural networks, have been applied to scleral images extracted from full-eye photographs, representing one of the earliest image-based explorations of non-invasive PCOS prediction (17). In another study, Wang Wei et al. demonstrated that machine learning algorithms—such as support vector machines, multilayer perceptrons, and eXtreme Gradient Boosting—combined with tongue and pulse characteristics, can accurately identify both early-stage and established PCOS (18). These findings highlight the promise of AI in leveraging objective, external physical features for preliminary PCOS screening.

Beyond imaging-based approaches, electronic health records (EHRs) are becoming an important resource for AI-driven PCOS diagnosis. Clinical symptoms combined with anti-Müllerian hormone (AMH) levels have been shown to be strong diagnostic indicators (19). Studies integrating machine learning with laboratory profiles—such as LH/FSH ratio, progesterone, AMH, and lipid panels—or with a minimal set of three clinical markers (LH, LH/FSH ratio, and AMH), have demonstrated substantial improvements in diagnostic accuracy (20–22). These efforts reflect a broader trend toward developing data-driven, multi-level diagnostic strategies capable of supporting both screening and confirmation of PCOS.

Patient history and physical examination remain the cornerstone of differential diagnosis. Key features typically encompass obstetric history (e.g., number of abortions, pregnancy status), lifestyle factors (e.g., exercise, fast-food consumption), clinical signs (e.g., weight gain, hirsutism), and specific gynecological metrics (e.g., menstrual cycle length, endometrial thickness, and follicle count). Leveraging such data, Zad Z et al. (23) developed machine learning models for PCOS prediction using electronic health records (EHRs) from the SafetyNet system. Similarly, Wang CY et al. (24) proposed the ‘mach-L’ prediction model based on a Taiwanese hospital cohort, identifying six key risk factors—age, triglycerides (TG), alanine aminotransferase (ALT/GPT), white blood cell count (WBC), uric acid (UA), and platelet count (Plt)—as the most predictive features.

Given the diagnostic significance of both ultrasonographically identified polycystic ovarian morphology (PCOM) and clinically relevant biomarkers from patient history and laboratory tests, researchers have increasingly integrated these multi-modal data sources for PCOS prediction. Building on this strategy, Abrar Alamoudi et al. (25) and Shanmugavadivel K et al. (26) applied deep learning and AI-based computational models to hybrid datasets that combine radiological imaging and laboratory test data, highlighting the enhanced potential of multi-modality fusion in PCOS diagnosis.

While some studies rely on proprietary hospital datasets, practical challenges in data collection make publicly available datasets a valuable alternative. In recent years, two PCOS datasets on Kaggle—one containing 45 structured features and the other comprising ultrasound images—have been widely adopted by researchers. Several studies have leveraged these resources to advance predictive modeling. For example, Sayma Alam Suha et al. (27) explored various feature subsets and machine learning models, ultimately proposing an optimized stacking ensemble that achieved superior performance using 25 principal components with a Gradient Boosting meta-learner. Similarly, Vairachilai S et al. (28) developed a hybrid approach combining SMOTE and logistic regression to predict PCOS based on demographic, clinical, and biochemical parameters.

Building on prior work, Rumman Ahmad et al. (29) proposed lightweight deep learning architectures—including LSTM, CNN, and hybrid CNN-LSTM models—augmented with SMOTE for data balancing. In parallel, Umaa Mahesswari G et al. (30) developed a two-tier Random Forest ensemble as an explainable AI (XAI) predictor, following extensive comparative analyses. Notably, Angela Zigarelli et al. (31) designed self-diagnostic prediction models for both prospective patients and clinicians, incorporating subgroup analyses that distinguish lean and obese PCOS phenotypes, consistent with their distinct biochemical, hormonal, and metabolic profiles (32).

Beyond purely data-driven modeling, research has increasingly focused on enhancing user accessibility and support in the PCOS domain. For instance, Rodriguez EM et al. (33) created a mobile health application that generates a PCOS risk score, providing a practical tool for initial self-assessment. Concurrently, studies have leveraged social media platforms, such as Reddit, to analyze patient discussions, offering insights into patient perspectives and enabling validation of user-reported clinical information (34, 35).

The advent of large language models (LLMs) like ChatGPT has opened further opportunities for exploration. Recent investigations have begun evaluating these AI tools for delivering reliable nutritional advice (36), general healthcare guidance for PCOS (37, 38), and even infertility treatment strategies, highlighting their potential as scalable sources of information and patient support.

Although researchers such as Zigarelli et al. have developed self-diagnostic models based solely on clinical features, and others have explored predictions using minimal feature sets, most existing feature-subset selection methods remain primarily statistically driven. Current approaches rely heavily on feature-importance rankings and often overlook key practical factors such as the source of each feature, the timing of data acquisition within clinical workflows, and—crucially—the economic and procedural burdens associated with obtaining those features.

PCOS diagnosis inherently involves multi-modal data. Prior multimodal studies have often emphasized modality-specific encoders or deep fusion architectures that combine imaging and structured data to enhance predictive performance. In contrast, the present study adopts a pragmatic feature-level integration strategy. Multimodal inputs are concatenated at the feature level without introducing modality-specific encoders or proposing a novel fusion algorithm. The term “multimodal” in this work therefore refers to the structured integration of heterogeneous clinical data sources rather than to the development of new representation-learning pipelines.

Instead of focusing on methodological novelty in multimodal fusion, our contribution lies in the design of a structured, stepwise modeling framework that explicitly incorporates data provenance, clinical workflow timing, and economic considerations. From a practical perspective, PCOS-related variables can be categorized by both source and cost:

- Clinical features (e.g., BMI, menstrual irregularity, hirsutism) are easily accessible.
- Laboratory test features (e.g., AMH, testosterone) require blood draws, an invasive procedure whose cost and patient discomfort typically increase with the number of tests ordered.
- Ultrasound features (e.g., antral follicle count) involve non-invasive imaging but incur higher costs.

Consequently, women facing financial constraints require diagnostic pathways that minimize laboratory expenditures, while those reluctant to undergo invasive procedures may prefer ultrasound-based approaches. These real-world considerations highlight the need for flexible, multi-tiered prediction models capable of providing accurate assessments across different levels of data availability, thereby narrowing the gap between algorithmic performance and clinical utility.

Accordingly, this study aims to develop a flexible PCOS prediction framework that accommodates diverse patient needs by enabling adaptable combinations of data sources. Our feature selection strategy innovatively incorporates data provenance, categorizing and prioritizing features into three types: clinical, laboratory test, and ultrasound features. Accordingly, we have constructed multiple dedicated models to support different clinical scenarios:

- Clinical model
- Clinical and laboratory test model
- Clinical and ultrasound model
- Multimodal model (clinical, laboratory test, and ultrasound)

This stepwise design emphasizes practical clinical applicability and resource-aware decision support rather than the introduction of a new multimodal fusion algorithm, and the framework is intentionally designed to be universally accessible. Potential patients can obtain an initial risk assessment using only clinical information and later receive refined evaluations once laboratory or imaging results become available, including by uploading photos of reports or ultrasound images. For healthcare providers, the system offers diagnostic support based on whatever data are currently accessible. For instance, if laboratory tests are completed but ultrasound is delayed, clinicians can still generate a preliminary assessment using the “clinical + laboratory test” model, enabling earlier decision-making and potentially expediting treatment.

To further enhance usability and clinical utility, we will develop a PCOS-specific intelligent agent that leverages the core prediction models to deliver knowledge-based support for diagnosis, management, and treatment decision-making.

## 2 Materials and methods

### 2.1 Data

In this retrospective study, we utilized an open-source Kaggle dataset comprising 45 feature fields (39). The data were accessed for research purposes on May 15, 2025. The dataset is fully de-identified, and the authors had no access to information capable of identifying individual participants at any stage of the study.

The dataset includes 541 female participants aged 20–48 years, of whom 177 were diagnosed with PCOS and 364 were not. From the original 45 columns, three irrelevant fields were excluded, resulting in 42 valid columns: 41 effective features and one PCOS label. Based on data source, the 41 features were categorized into three major types: clinical features, laboratory test features, and ultrasound features. Detailed feature groupings are presented in **Table 1**.

**Table 1:** Features details.

Feature type	Feature names	Numbers
Clinical	**Body features:** BM, Height, Hip, Waist, Waist/Hip, Weight **Symptomatic features:** Skin darkening, Pimples, Hair loss, Hair growth, Cycle, Cycle Length, Weight gain **Statistical features:** Age, Blood group, Marriage status, Abortions, Fast food, Pregnant, Reg.Exercise **External measurement features:** BP_Systolic, BP_Diastolic, RR, Pulse rate	24
Laboratory test	AMH, FSH, FSH/LH, Hb, I beta-HCG, II beta-HCG, LH, PRG, PRL, RBS, TSH, Vit D3	12
Ultrasound	Follicle No. (L), Follicle No. (R), Avg. F size (L), Avg. F size (R), Endometrium	5

Bold values indicate the best performance in each column.

The clinical features comprise 24 items, further subdivided into four categories: body, symptomatic, statistical, and external measurement features.

- Body features: BMI, height, hip circumference, waist circumference, waist-hip ratio, and weight.
- Symptomatic features: skin darkening, pimples, hair loss, hirsutism, menstrual cycle, cycle length, and weight gain.
- Statistical features: age, blood group, marital status, number of abortions, fast-food consumption, pregnancy status, and regular exercise.
- External measurement features: systolic blood pressure, diastolic blood pressure, respiratory rate, and pulse rate.
- The laboratory test features include 12 items: AMH, follicle-stimulating hormone (FSH), FSH-to-LH ratio, glycated hemoglobin, human chorionic gonadotropin I (hCG I), human chorionic gonadotropin II (hCG II), luteinizing hormone (LH), progesterone, prolactin, random blood sugar, thyroid-stimulating hormone (TSH), and vitamin D3.
- The ultrasound features consist of 5 items: number of antral follicles in the left ovary, number of antral follicles in the right ovary, average follicle size in the left ovary, average follicle size in the right ovary, and endometrial thickness.

### 2.2 Overview of statistical analysis

In this study, a structured multimodal learning framework was adopted, in which heterogeneous clinical data sources were treated as distinct modalities. Specifically, three modalities were defined: (1) clinical and demographic features, (2) laboratory test features, and (3) ultrasound-derived features. Multimodal integration was implemented at the feature level by concatenating modality-specific feature subsets into a unified input space. Different models were constructed using single-modality and multi-modality combinations to systematically evaluate the contribution of each modality and their joint effects on prediction performance. Although the proposed framework does not involve modality-specific encoders, the multimodal concept here refers to the integration of heterogeneous clinical data sources rather than different data acquisition technologies.

**Figure 1:**
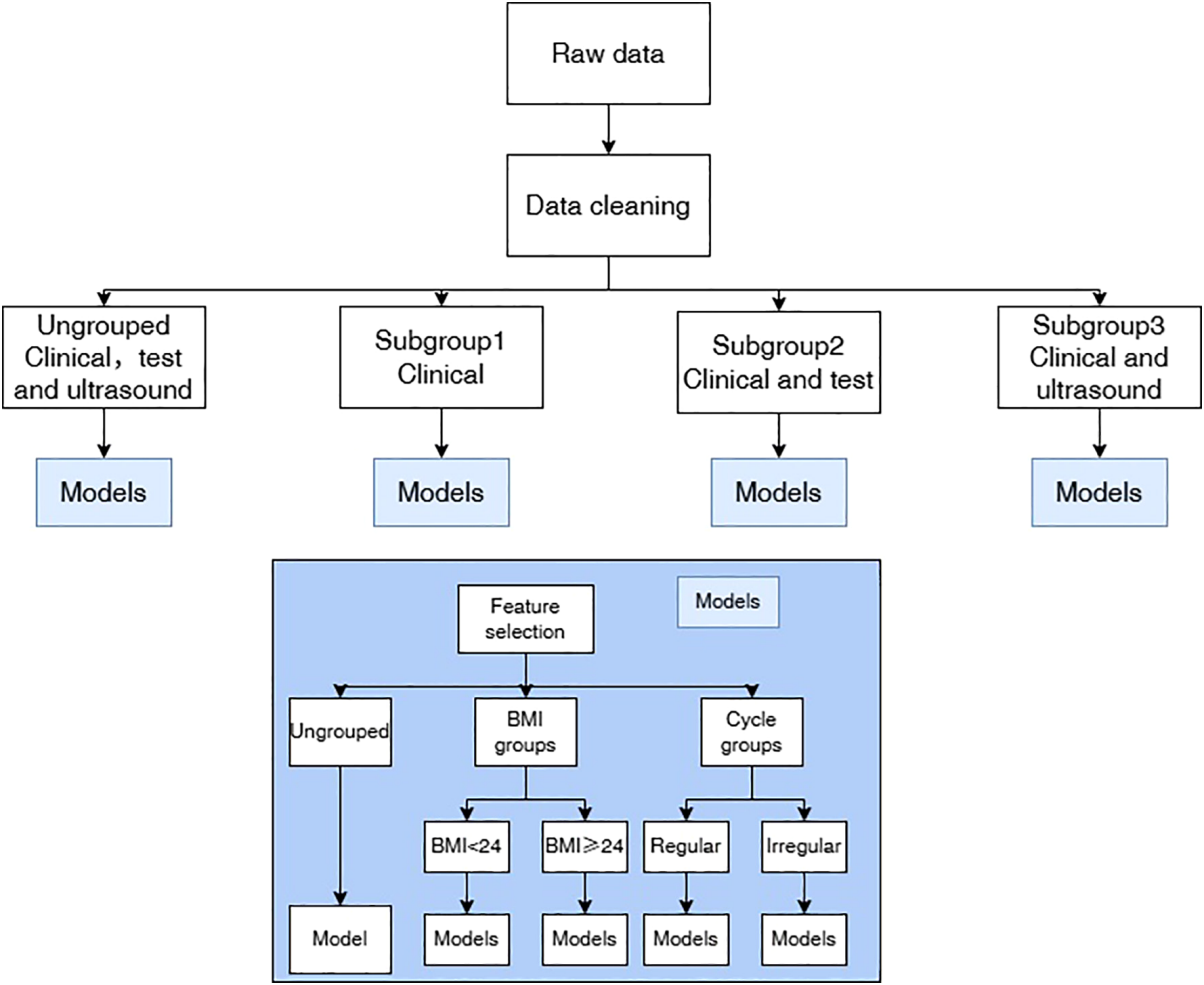
Flowchart of models and subgroup study.

We developed four predictive models: the Clinical model, the Clinical + Laboratory model, the Clinical + Ultrasound model, and the Multimodal model (incorporating clinical, laboratory test, and ultrasound features). For example, the clinical and laboratory test model relies exclusively on clinical and laboratory test features. During model development, we performed feature importance and correlation analyses stratified by data source (clinical, laboratory test, and ultrasound). Additionally, given that BMI and menstrual irregularity are key PCOS characteristics often associated with distinct PCOS subtypes, we conducted subgroup analyses based on these features to examine their influence on model performance. The detailed model development workflow is illustrated in **Figure 1**.

For correlation analysis, we primarily employed Pearson correlation coefficients to identify features strongly associated with PCOS diagnosis while excluding irrelevant ones, thereby reducing feature redundancy. The Pearson correlation coefficient $r_{xy}$ is defined in Equation 1:

(1) $$r_{xy}=\frac{\sum_{i=1}^{n}\left(x_{i}-\bar{x})(y_{i}-\bar{y}\right)}{\sqrt{\sum_{i=1}^{n}{\left(x_{i}-\bar{x}\right)}^{2}}\sqrt{\sum_{i=1}^{n}{\left(y_{i}-\bar{y}\right)}^{2}}}$$

Where $x_{i}$and $y_{i}$ represent the values of feature x and the PCOS label y for sample i, and $\bar{x}$ and $\bar{y}$ denote their respective means. Features with $|r_{xy}|<0.15\;$ werewere excluded from modeling to reduce noise while retaining predictors with meaningful association to PCOS. For example, among clinical features, external measurements such as systolic and diastolic blood pressure showed negligible correlation with PCOS.

Although feature-correlation and importance analyses help filter irrelevant variables and retain key predictors, real-world patients often prefer simpler and more cost-efficient approaches to PCOS assessment. Self-evaluation using only basic clinical information—without blood tests or ultrasound—can be particularly valuable. Individuals identified as high risk through such preliminary screening can then seek medical consultation, enabling earlier detection. Clinicians may subsequently recommend laboratory tests or ultrasound examinations tailored to the patient’s specific presentation.

To reduce patients’ financial burden and improve the efficient use of healthcare resources, it is essential to achieve accurate PCOS diagnosis with minimal reliance on laboratory testing. Many assays—such as progesterone and other sex hormone measurements—cannot be completed within the same menstrual cycle, often delaying the availability of a full diagnostic panel; thus, waiting for all test results is clinically impractical. Moreover, certain tests, including blood glucose assessments, may require additional blood draws that are unnecessary for some patients and may increase psychological discomfort. For individuals reluctant to undergo invasive procedures, ultrasound imaging offers a suitable non-invasive alternative.

Accordingly, we argue that feature-importance evaluation should incorporate the source of each variable—clinical information, laboratory tests, and ultrasound findings. When selecting features based on importance, considering their provenance allows the development of models tailored to different data combinations, thereby accommodating the diverse needs of the population. The following sections detail the feature analysis procedures and machine learning techniques used in this study.

Random Forest is an efficient ensemble learning algorithm (40) capable of performing both classification and regression tasks by constructing multiple decision trees and aggregating their predictions. Its core principle is based on the Bagging integration strategy combined with the introduction of feature randomness, which enhances the model’s generalization ability and robustness. The algorithm consists of two main steps. First, bootstrap sampling is used to generate multiple training subsets from the original dataset. Second, during node splitting, each decision tree considers only a randomly selected subset of features when determining the optimal split. This dual-randomness design increases model diversity and reduces the overfitting tendency of individual trees. For classification tasks, final predictions are obtained through majority voting across all trees.

Owing to its high accuracy, robustness to outliers, capacity to handle high-dimensional data, and ability to evaluate feature importance, Random Forest has been widely applied across various domains. Given its favorable performance in previous PCOS-related studies and its suitability for our research objectives, we selected Random Forest as the primary machine learning algorithm for this study.

To prevent overfitting or underfitting of the classification model, the dataset was randomly split, with 70% of the samples used for model training and the remaining 30% reserved for validation and performance evaluation.

All preprocessing steps were performed exclusively on the training data, and the derived parameters were subsequently applied to the validation set to avoid information leakage. Specifically, categorical variables (e.g., blood group, marital status) were encoded using one-hot encoding, and continuous variables (e.g., BMI, hormone levels, follicle counts) were standardized to zero mean and unit variance. Missing values were imputed using the median for continuous features and the mode for categorical features. Class imbalance was addressed by applying class weights during Random Forest training; no oversampling or synthetic sampling methods were used.

Model performance was assessed using four evaluation metrics: accuracy, sensitivity (recall), precision, and F1-score. Accuracy is the most intuitive and commonly used metric, reflecting the proportion of correctly classified samples among all observations. Sensitivity (recall) measures the proportion of true positives correctly identified, while precision indicates the proportion of true positives among all positive predictions. The F1-score, defined as the harmonic mean of precision and recall, summarizes the trade-off between false-positive and false-negative errors. In imbalanced datasets, the F1-score typically provides a more informative evaluation than accuracy alone.

## 3 Results

### 3.1 Performance of machine learning

To evaluate the performance of models based on different combinations of feature sources, we conducted feature importance assessment and selection for various feature groupings. For clarity in subsequent analyses, we defined the following subgroups:

- Subgroup 1: Clinical data only
- Subgroup 2: Clinical and laboratory test data
- Subgroup 3: Clinical and ultrasound data
- Ungrouped: Clinical, laboratory test, and ultrasound data

**Figure 2:**
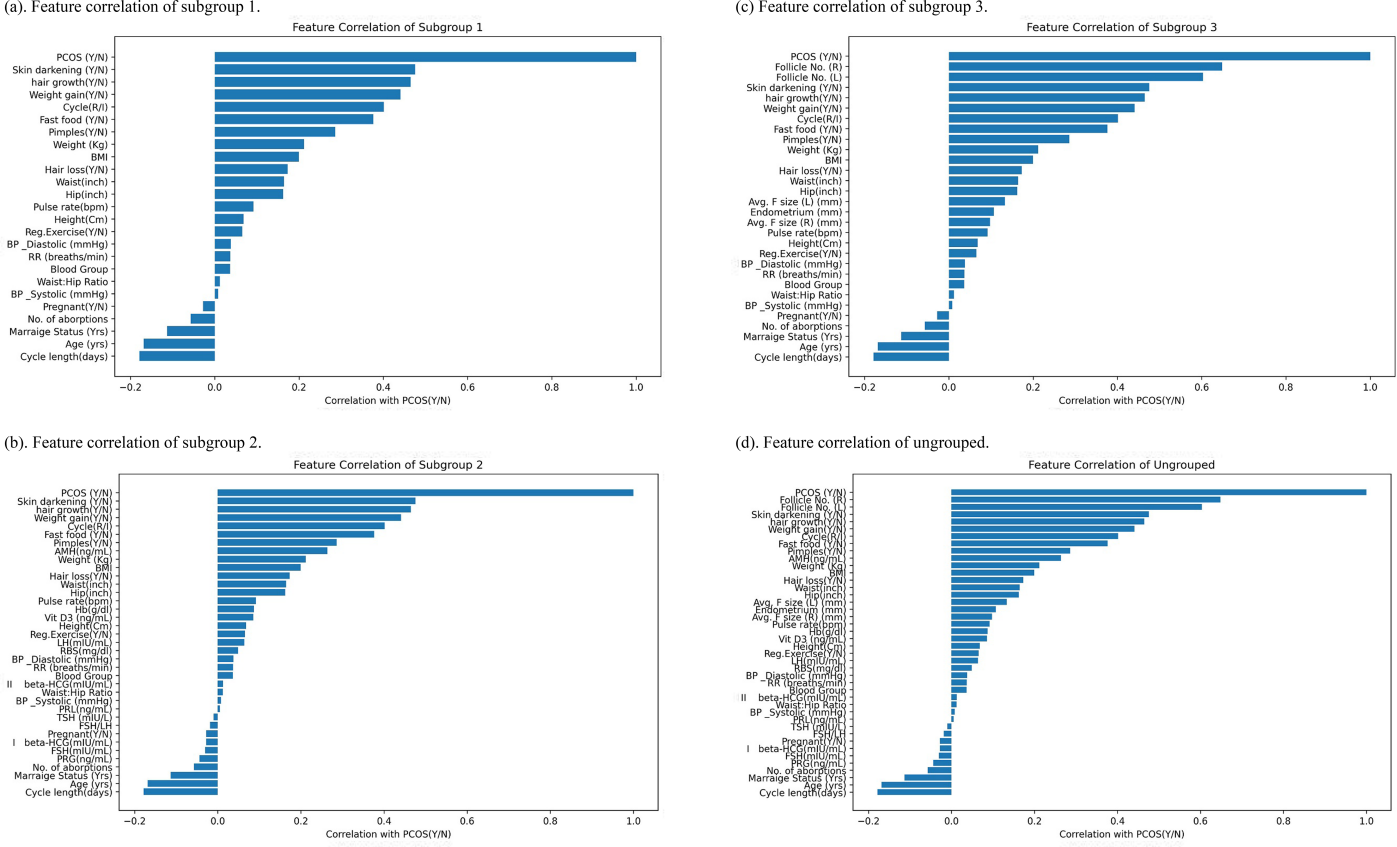
Feature correlation plots. **(a)** Feature correlation of subgroup 1. **(b)** Feature correlation of subgroup 2. **(c)** Feature correlation of subgroup 3. **(d)** Feature correlation of ungrouped.

Notably, all subgroups include clinical features, as these are the most readily accessible. Pearson correlation analyses were conducted within each subgroup, and the resulting correlation heatmaps are presented in **Figure 2**.

As shown, the four external measurement variables—systolic blood pressure, diastolic blood pressure, respiratory rate, and pulse rate—displayed minimal correlation with PCOS. Among the clinical features, BMI, irregular menstrual cycles, skin darkening, and hair growth showed the strongest associations with the condition. These results are consistent with previous findings and align with key components of the Rotterdam criteria, including oligo-anovulation and hyperandrogenism. For laboratory tests, AMH exhibited the highest correlation with PCOS, whereas for ultrasound features, antral follicle counts in both the left and right ovaries were most strongly associated.

Features with a correlation coefficient greater than 0.15 with PCOS were selected for each subgroup. The selected features are summarized in **Table 2**. Subgroup 1 comprises 13 clinical features. Subgroup 2 includes 14 features, consisting of the 13 clinical features plus one laboratory test feature (AMH). Subgroup 3 contains 15 features, comprising the 13 clinical features and two ultrasound features (number of antral follicles in the left and right ovaries). The ungrouped set consists of 16 features, including the 13 clinical features, the laboratory test feature AMH, and the two ultrasound features.

**Table 2:** Feature selection for different groups.

Models	Groups	Feature numbers	Feature selection	Feature selection numbers
Clinical	Subgroup 1	24	**Body features:**BMI, Waist, Hip, Weight **Symptomatic features:**Skin darkening, Pimples, Hair loss, Hair growth, Cycle, Cycle Length, Weight gain **Statistical features:**Age, Fast food	13
Clinical and laboratorytest	Subgroup 2	36	**Body features:**BMI, Waist, Hip, Weight **Symptomatic features:**Skin darkening, Pimples, Hair loss, Hair growth, Cycle, Cycle Length, Weight gain **Statistical features:**Age, Fast food **Test feature:**AMH	14
Clinical and ultrasound	Subgroup 3	29	**Body features:**BMI, Waist, Hip, Weight **Symptomatic features:**Skin darkening, Pimples, Hair loss, Hair growth, Cycle, Cycle Length, Weight gain **Statistical features:**Age, Fast food **Ultrasound features:**Follicle No. (L), Follicle No. (R)	15
Multimodal	Ungrouped	41	**Body features:**BMI, Waist, Hip, Weight **Symptomatic features:**Skin darkening, Pimples, Hair loss, Hair growth, Cycle, Cycle Length, Weight gain **Statistical features:**Age, Fast food **Laboratory test feature:**AMH **Ultrasound features:**Follicle No. (L), Follicle No. (R)	16

Bold values indicate the best performance in each column.

Random Forest models were trained on different feature subgroups, and their predictive performance is summarized in **Table 3**. The ungrouped model, which incorporated all available features, achieved the highest overall performance with an accuracy of 0.948. In contrast, Subgroup 1, relying solely on clinical features and containing the fewest input variables, showed the lowest performance among all subgroups, although it still achieved a respectable accuracy of 0.850. Comparison between Subgroup 2 and Subgroup 3 revealed superior predictive performance for the latter, highlighting the added value of incorporating ultrasound features alongside clinical data. Based on these findings, Random Forest was first employed to evaluate the incremental predictive value of different feature combinations (**Table 3**), followed by representative algorithm comparisons on selected feature configurations to determine the optimal modeling strategy (**Tables 4**, **5**). Consequently, Random Forest was adopted for all subsequent stratified analyses.

**Table 3:** Performance of models.

Models	Clinical	Clinical and laboratory test	Clinical and ultrasound	Multimodal
Accuracy	0.85	0.884	0.902	0.948
Precision	0.839	0.879	0.885	0.951
Recall	0.734	0.797	0.844	0.906
F1 score	0.783	0.836	0.864	0.928

**Table 4:** Model performance comparison on Subgroup 1 (clinical features).

Models	Accuracy	Precision	Recall	F1-score
Random Forest	0.85	0.839	0.734	0.783
Logistic Regression	0.802	0.788	0.728	0.757
Support Vector Machine	0.818	0.815	0.715	0.762
Gradient Boosting	0.832	0.830	0.727	0.775

**Table 5:** Model performance comparison on the ungrouped feature set (all features).

Models	Accuracy	Precision	Recall	F1-score
Random Forest	0.948	0.951	0.906	0.928
Logistic Regression	0.915	0.905	0.878	0.891
Support Vector Machine	0.925	0.928	0.865	0.895
Gradient Boosting	0.938	0.940	0.886	0.912

To avoid redundant presentation and to maintain clarity, comparative evaluations across multiple machine learning algorithms were conducted only on two representative feature configurations: the minimal-feature setting (Subgroup 1, clinical features only) and the full-feature setting (ungrouped, incorporating clinical, laboratory, and ultrasound features).

As shown in **Tables 4** and **5**, Random Forest consistently outperformed Logistic Regression, Support Vector Machine, and Gradient Boosting across all evaluated metrics in both settings. Given this consistent superiority, Random Forest was selected as the primary modeling approach. Consequently, all subsequent subgroup analyses and stratified evaluations (**Tables 6**–**9**) were performed exclusively using Random Forest models.

**Table 6:** Performance of subgroup 1 models.

Models	BMI<24	BMI>=24	Irregular cycle	Regular cycle
Numbers	78	85	47	116
Accuracy	0.897	0.777	0.787	0.853
Precision	0.75	0.75	0.929	0.571
Recall	0.75	0.73	0.765	0.6
F1 score	0.75	0.74	0.839	0.585

**Table 7:** Performance of subgroup 2 models.

Models	BMI<24	BMI>=24	Irregular cycle	Regular cycle
Numbers	78	85	47	116
Accuracy	0.923	0.824	0.851	0.888
Precision	0.813	0.806	0.936	0.684
Recall	0.813	0.784	0.853	0.65
F1 score	0.813	0.795	0.892	0.667

**Table 8:** Performance of subgroup 3 models.

Models	BMI<24	BMI>=24	Irregular cycle	Regular cycle
Numbers	78	85	47	116
Accuracy	0.935	0.859	0.851	0.897
Precision	0.867	0.857	1	0.7
Recall	0.813	0.811	0.794	0.7
F1 score	0.839	0.833	0.885	0.7

**Table 9:** Performance of ungrouped models.

Models	BMI<24	BMI>=24	Irregular cycle	Regular cycle
Numbers	78	85	47	116
Accuracy	0.948	0.882	0.872	0.914
Precision	0.875	0.865	0.967	0.727
Recall	0.875	0.865	0.853	0.8
F1 score	0.875	0.865	0.906	0.762

Given the significant influence of BMI and menstrual irregularity, we further evaluated model performance across different feature subgroups under varying BMI and menstrual cycle conditions, as summarized in **Tables 6**-**9**. Participants were stratified into a high-BMI group (BMI **>=** 24) and a normal-BMI group (BMI < 24). Results indicate that model performance was consistently slightly lower in the high-BMI group across all feature subsets, which may be attributable to the higher likelihood of comorbidities in women with elevated BMI, complicating accurate PCOS prediction.

Furthermore, when participants were grouped based on the presence or absence of menstrual irregularity, precision was consistently higher in the subgroup with irregular cycles. Notably, even in Subgroup 1, precision reached 0.929 within this population, highlighting the predictive value of menstrual irregularity for PCOS detection.

### 3.2 Performance of large language model

To develop an intelligent PCOS agent capable of providing diagnostic and therapeutic guidance through question answering, the integration of a large language model (LLM) is essential. Several widely adopted general-purpose LLMs are currently available, such as ChatGPT-5 (41). In the medical domain, notable general-purpose models include MedGemma (42) and LLaVA-Med (43). Domestically, recent models such as Lingshu (44) also represent advanced general-purpose medical LLMs. Our approach involves comparing the performance of these models to identify a suitable LLM for integration into our system.

Prior studies have evaluated the use of ChatGPT-4 for PCOS-related question answering (45). Building on this work, we employed the question-answering dataset constructed by Irmak Gunesli et al. (37), which is based on the Recommendations from the 2023 International Evidence-based Guideline for the Assessment and Management of Polycystic Ovary Syndrome (46). The dataset contains 44 guideline-based questions. To ensure consistency with the intended Chinese-language application scenario, all questions were translated into Chinese prior to evaluation. Using this dataset, we assessed the performance of advanced LLMs—including ChatGPT-5 and specialized medical models such as Lingshu—in answering PCOS-related questions. A standardized prompt template was applied across all models, asking the model to answer each question based on clinical guideline knowledge, instructing them to provide concise, guideline-based responses to each clinical question. The goal was to identify a model that could be effectively integrated into our intelligent PCOS agent for knowledge-based queries without the need for additional fine-tuning.

The performance of LLMs was evaluated using a 6-point Likert scale across two dimensions: accuracy and completeness. Model responses were classified into three tiers: scores of 1–2 indicated inaccurate answers, 3–4 represented generally accurate responses, and 5–6 denoted highly accurate answers. Evaluations were conducted by two physicians specializing in PCOS. The final score for each response was calculated as the average of the two evaluators’ ratings. To minimize bias, the evaluators were presented only with the questions and model responses, with all model identifiers anonymized during the assessment process.

We conducted a comparative analysis of the latest general-purpose LLM, ChatGPT-5, alongside several medical-specialized models, including Lingshu, MedGemma, and LLaVA-Med. The evaluation results are summarized in **Table 10**. Considering that the intended application of our system targets Chinese-language clinical consultation scenarios, evaluating model responses in Chinese provides a more realistic assessment of practical usability. Under this setting, the Lingshu model demonstrated superior performance in both accuracy and completeness. Accordingly, we selected Lingshu as the foundational LLM for our PCOS-specific intelligent agent, AutoPCOS.

**Table 10:** Performance of large language models.

Models	Lingshu	MedGemma	LLaVA-Med	ChatGPT5
Accuracy	5.0 ± 1.0	4.8 ± 1.2	4.6 ± 1.3	4.9 ± 1
Completeness	3.5 ± 0.8	3.3 ± 0.9	3.1 ± 1.1	3.1 ± 1.1

Scores are presented as mean ± standard deviation (SD).

### 3.3 PCOS agent

To enhance user accessibility, we have integrated our multi-subgroup models into an intelligent agent AutoPCOS built on the Dify platform. This agent incorporates not only the various PCOS diagnostic models but also a custom-built PCOS knowledge base. For the LLM component, we adopted Alibaba’s recently released Lingshu model.

**Figure 3:**
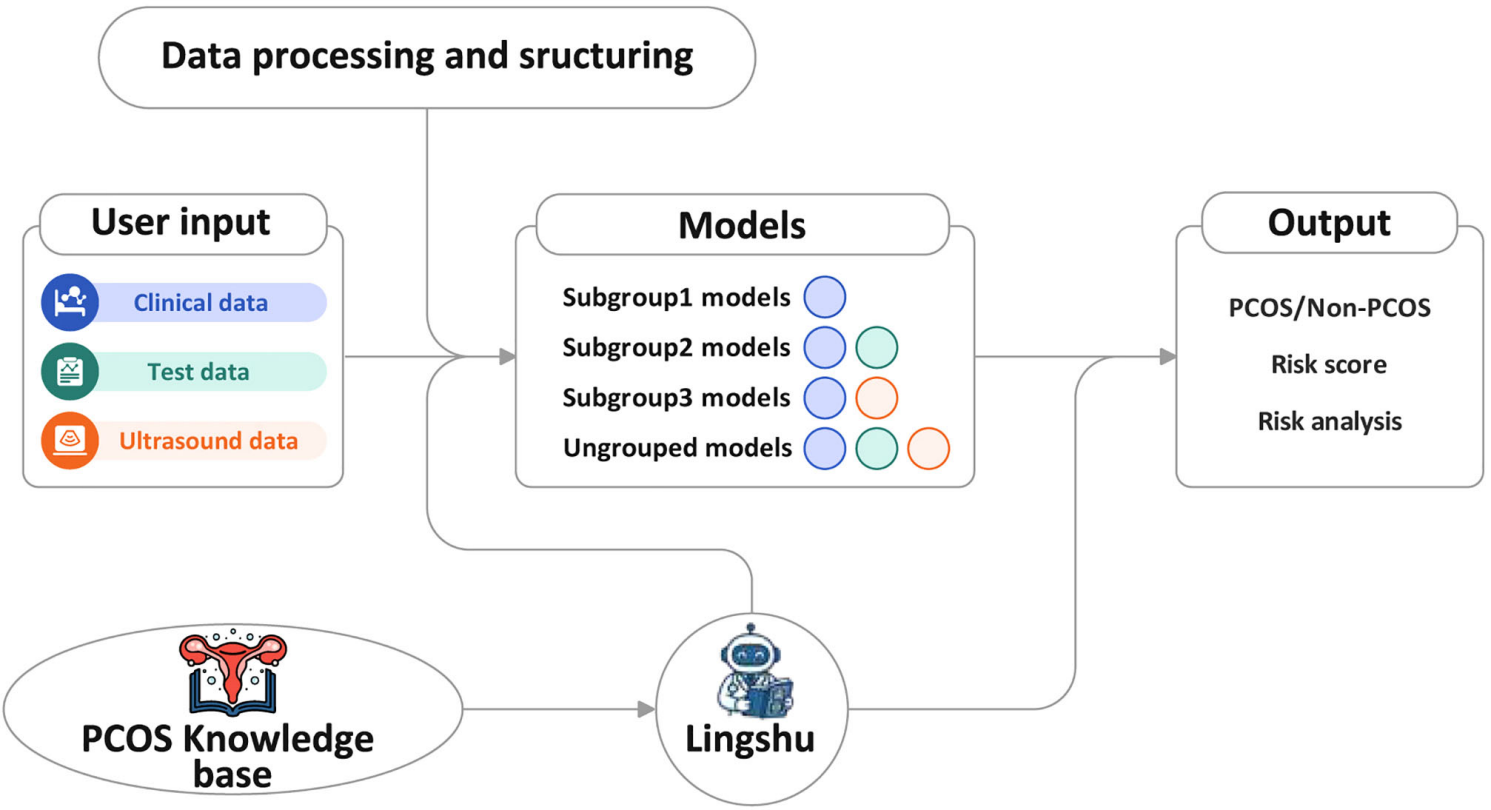
Agent workflow.

Users can input their clinical information as prompted on the interface. The agent then generates a PCOS risk estimate based on the predictive models and, leveraging the knowledge base and the LLM, provides explanatory information to support user understanding and clinical consultation (see **Figure 3**). A PCOS risk probability ≥50% indicates higher risk, while <50% indicates lower risk. The output is not intended to replace clinical diagnosis.

The custom analysis module is designed to support external validation with user-provided datasets. Prior to analysis, all missing values are automatically imputed, after which users may download both the imputed dataset and the corresponding prediction results for local research and clinical use.

**Figure 4:**
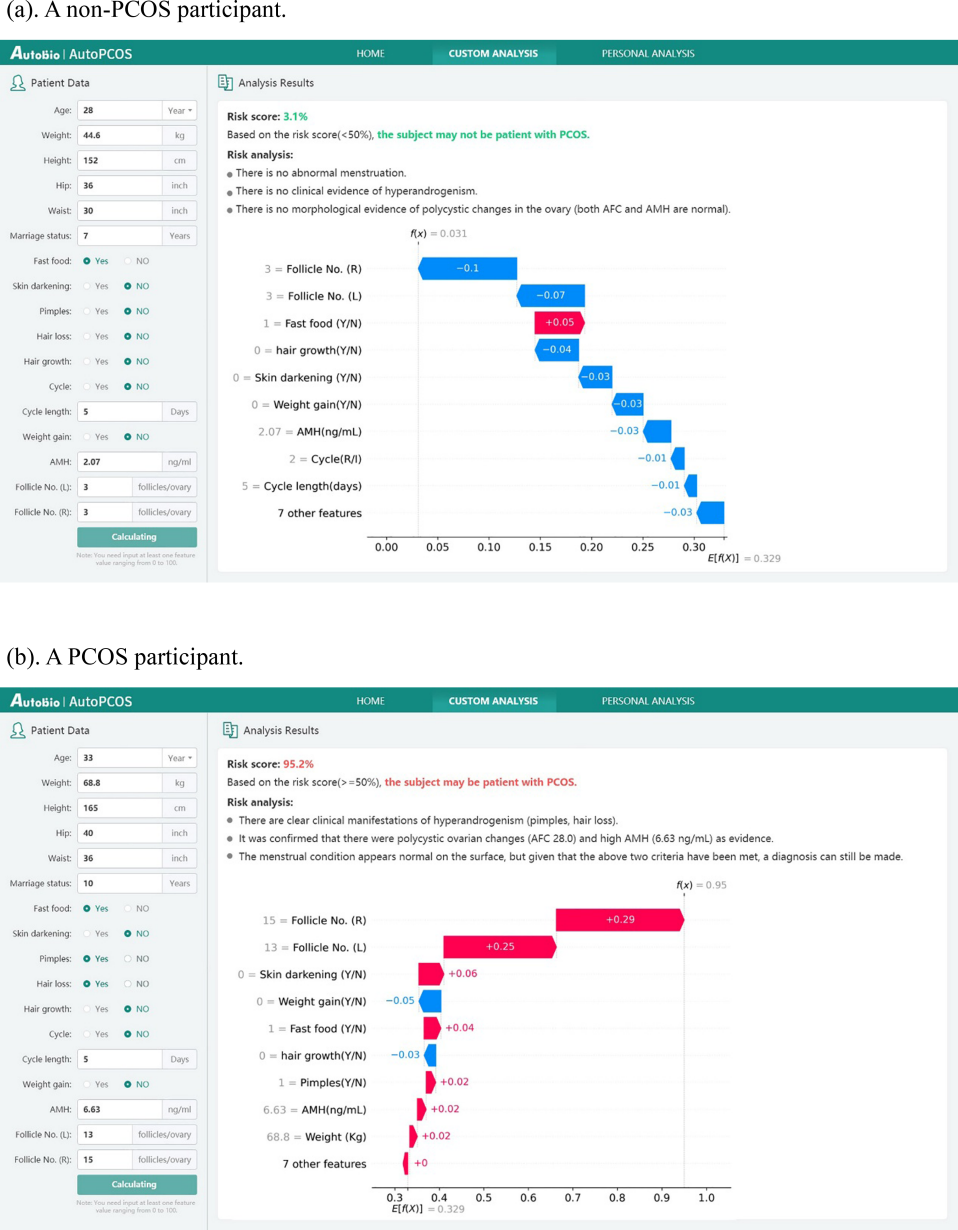
The risk scores of PCOS in two participants were calculated using the web application. **(a)** A non-PCOS participant. **(b)** A PCOS participant.

For individual predictions, users can input any available feature values (at least one feature is required) and click the “Calculate” button. The agent then automatically generates a risk assessment. As shown in **Figure 4**, the output provides not only the predicted PCOS risk probability but also a detailed risk interpretation, supplemented with a SHAP (47) visualization that illustrates the contribution of each feature to the prediction. Red bars indicate features that shift the prediction toward PCOS, whereas blue bars indicate those that shift it toward Non-PCOS. If the entered information is insufficient for reliable inference, the system prompts the user to provide additional features to refine the prediction.

Our intelligent agent AutoPCOS supports multiple flexible input modalities. Users may either describe their clinical features in free-text form or upload images of laboratory reports and ultrasound examination sheets. Uploaded reports are processed using the PaddleOCR framework for text recognition, followed by rule-based extraction of predefined laboratory indicators relevant to PCOS assessment. All extracted values are presented to users for verification prior to model inference, and basic range-validation rules are applied to flag implausible entries, reducing the risk of mis-extraction. The system then automatically selects the appropriate diagnostic model to generate an individualized PCOS risk prediction.

## 4 Discussion

PCOS is a common hormonal disorder affecting women, yet its identification and diagnosis remain challenging due to the condition’s clinical complexity, even when standardized criteria and diagnostic tests are available. These challenges are further amplified for women with limited financial resources, who often encounter barriers in accessing reliable PCOS-related information. Providing a convenient and accessible information gateway can therefore play a crucial role in supporting patient education, risk awareness, and informed clinical consultation in the context of PCOS.

The objective of this study is to develop an intelligent agent system that integrates multiple functions—including PCOS risk prediction, diagnostic support, and knowledge-based question answering. The system is built upon two key contributions: first, the construction of a multimodal PCOS diagnostic model, and second, the development of a PCOS-focused diagnostic and consultation agent powered by the Lingshu large language model. The evaluation of the large language model component was designed as an exploratory assessment of guideline consistency and response quality, rather than a formal benchmark comparison across different models.

Based on a dataset of 541 female participants, we grouped all variables into three major categories according to their sources: clinical features, laboratory test features, and ultrasound features. Using these inputs, we developed four predictive models: the Clinical model, the Clinical + Laboratory model, the Clinical + Ultrasound model, and the Multimodal model integrating all three feature types. This multi-model framework offers users flexible and cost-effective options for PCOS risk prediction.

In feature selection, Pearson correlation analysis indicated that systolic and diastolic blood pressure were minimally correlated with PCOS. Among laboratory tests, anti-Müllerian hormone (AMH) emerged as the most strongly associated biomarker in this dataset. While AMH demonstrated substantial predictive value, it should not be interpreted as a standalone diagnostic test. In accordance with the 2023 International Evidence-based Guideline for PCOS, AMH may serve as an adjunctive indicator—particularly when ultrasound access is limited—but formal diagnosis should remain based on established diagnostic criteria. For ultrasound features, the number of antral follicles in both the left and right ovaries was identified as the most relevant indicator.

The Random Forest classifier was chosen for model training and evaluation. Model performance was assessed using precision, recall, accuracy, and F1-score. The Multimodal model, incorporating clinical, laboratory, and ultrasound features, achieved the highest accuracy, reflecting its use of the most complete feature set. Among the partial models, the “clinical + ultrasound” model outperformed the “clinical + laboratory test” model overall. Although the “clinical” model showed the lowest overall performance, its ability to predict PCOS with 85% accuracy using only clinical features still provides practical value for initial user assessment.

We further evaluated model performance in subgroups defined by BMI and menstrual regularity. Menstrual irregularity emerged as a significant risk factor for PCOS, with models demonstrating higher predictive accuracy for participants with irregular cycles. These results indicate that the proposed framework can help reveal patterns linked to elevated PCOS risk in specific subgroups.

### 4.1 Comparison with Kaggle-based ensemble models

Given that this study utilizes the same publicly available Kaggle PCOS dataset as several previous machine learning studies, it is important to interpret the present findings in the context of existing literature. Several Kaggle-based studies have reported strong predictive performance using tuned ensemble or stacking approaches, such as the modified stacking framework proposed by Suha et al. (Heliyon, 2023), which reported an accuracy of 95.7%.

However, conducting a strict head-to-head comparison with these studies is challenging due to limited reproducibility of the original experimental settings. In particular, the exact data partition strategies and detailed preprocessing pipelines are not fully specified, and publicly available implementation details remain limited, making strict replication difficult.

Moreover, differences in feature engineering and preprocessing strategies may substantially influence reported performance. For instance, Suha et al. employed PCA-based dimensionality reduction to derive principal components, whereas the present study applied correlation-based feature filtering after stratified data splitting while preserving clinically interpretable variables grouped by data source.

In addition, the current study implemented a strict evaluation protocol in which all preprocessing procedures—including encoding, scaling, imputation, and feature selection—were performed exclusively on the training data and subsequently applied to validation data. Such procedures help reduce the risk of information leakage, which can otherwise lead to optimistic performance estimates in relatively small datasets such as the Kaggle PCOS dataset (n = 541).

Finally, it should be noted that the objectives of the present study differ from many previous Kaggle-based modeling studies. Rather than focusing solely on maximizing predictive accuracy through ensemble optimization, this work emphasizes the development of a structured multimodal framework designed to support stepwise PCOS risk assessment under different data availability scenarios. Therefore, differences in reported performance may reflect variations in modeling objectives, feature engineering strategies, and evaluation protocols rather than purely algorithmic superiority.

Future work may include fully reproducible benchmarking once standardized evaluation protocols or publicly available implementations become available.

### 4.2 Limitations and generalizability

Several limitations regarding the generalizability of this study should be acknowledged. First, the predictive models were developed and evaluated using an open-source dataset, which may not fully capture the heterogeneity of real-world clinical populations. The sample size was relatively limited, and all participants were female patients within a specific age range, which may restrict the generalizability of the findings to broader or more diverse populations.

Second, although multiple data sources were integrated, the dataset lacked longitudinal follow-up data and external validation cohorts, limiting the assessment of model performance across different clinical settings and healthcare systems.

Nevertheless, the primary objective of this study was to demonstrate the feasibility and potential clinical value of a structured multimodal PCOS diagnostic framework. To enhance robustness, generalizability, and clinical applicability, future work will focus on multi-center validation using real-world hospital data.

The PCOS intelligent agent AutoPCOS developed in this study provides an integrated platform that combines PCOS risk prediction with knowledge-based consultation. It offers users a highly accessible and cost-effective solution, providing not only PCOS risk predictions but also detailed risk analyses to support informed health decisions.

However, to avoid potential overdiagnosis and unnecessary psychological burden, it is important to clarify the intended scope of application. Importantly, this framework is not intended for population-level screening or for use in asymptomatic individuals; its purpose is to support risk assessment and diagnostic decision-making only in the presence of relevant clinical symptoms or clinical concern.

## 5 Conclusion

This study presents AutoPCOS, a stepwise multimodal intelligent framework for PCOS risk stratification and diagnostic support. By integrating three clearly defined modalities—clinical, laboratory, and ultrasound features—we developed four predictive models tailored to different levels of resource availability: the Clinical model, the Clinical + Laboratory model, the Clinical + Ultrasound model, and the Multimodal model. Comparative evaluation across several baseline classifiers confirmed the robustness of the Random Forest model within this framework. Subgroup analyses further demonstrated consistent predictive performance across BMI categories and menstrual-cycle patterns, supporting the value of subgroup-specific modeling. To enhance interpretability and user engagement, we incorporated a PCOS knowledge base and the Lingshu large language model, enabling intuitive risk explanations and personalized guidance.

While the proposed models show promise, their development using publicly available datasets limits generalizability. Future work will involve collaboration with clinical centers to obtain real-world patient data and specialized PCOS datasets for fine-tuning the language model, thereby improving the reliability and applicability of AutoPCOS in clinical practice. Overall, this framework offers a scalable, stepwise approach for preliminary PCOS risk assessment, risk assessment, and diagnostic support, bridging artificial intelligence with clinically informed decision support.

## Data Availability

Publicly available datasets were analyzed in this study. This data can be found here: https://www.kaggle.com/datasets/prasoonkottarathil/polycystic-ovary-syndrome-pcos.
